# Supplementary material for: Histo-Miner: Deep learning based tissue features extraction pipeline from H&E whole slide images of cutaneous squamous cell carcinoma
Source: PLoS Comput Biol. 2026 Jan 21;22(1):e1013907. doi: 10.1371/journal.pcbi.1013907 (PMC12854473; doi:10.1371/journal.pcbi.1013907)
Supplement: S1 Material — It includes the following sections:Poor precision in tumor and healthy epithelial detection from state-of-the-art pretrained models.SCC Hovernet loss functions.Hyperparameters grid search for SCC Segmenter.Probability of distance overestimation.List of all features from Tissue Analyser.Ranking of features after cross-validation with mRMR selection.ROC curves of the best-feature classifier across CV folds.Stain variability for the different cohorts of TumSeg dataset. (PDF) [file pcbi.1013907.s001.pdf]

Supplementary Data

Table of Contents

– Poor precision in tumor and healthy epithelial detection from state of the art pretrained models . . . 3
– SCC Hovernet loss functions . . . 3
– Hyperparameters grid search for SCC Segmenter . . . 3
– Probability of distance overestimation . . . 4
– List of All Features from Tissue Analyser . . . 10
– Ranking of Features after Cross-Validation with mRMR Selection . . . 10
– ROC curves of classifier with best kept features for all cross-validation folds . . . 11
– Stain variability for the different cohorts of TumSeg dataset . . . 11

Poor precision in tumor and healthy epithelial detection from state of the art pretrained models

We used Hovernet [1], CellViT-256 and CellVit-SAM-H [2] models pretrained on Pannuke dataset [3] to apply inference on 2 TumSeg samples where tumor regions are annotated by 2 experts. We show in **Fig A in S1 Material** that the models are not able to recognize healthy epithelial, and classify most of the epithelial cells outside tumor regions as tumor cells. On the other hand, the tumor region segmentation performed by SCC Segmenter, guiding cell classification in Histo-Miner, has an average accuracy mAcc = 0.969 and mean intersection over union mIoU = 0.907 on our test set. All cells previously classified as tumor by SCC Hovernet that are outside tumor regions segmented by SCC Segmenter will be re-classified as non-neoplastic epithelial cells during Histo-Miner processing.

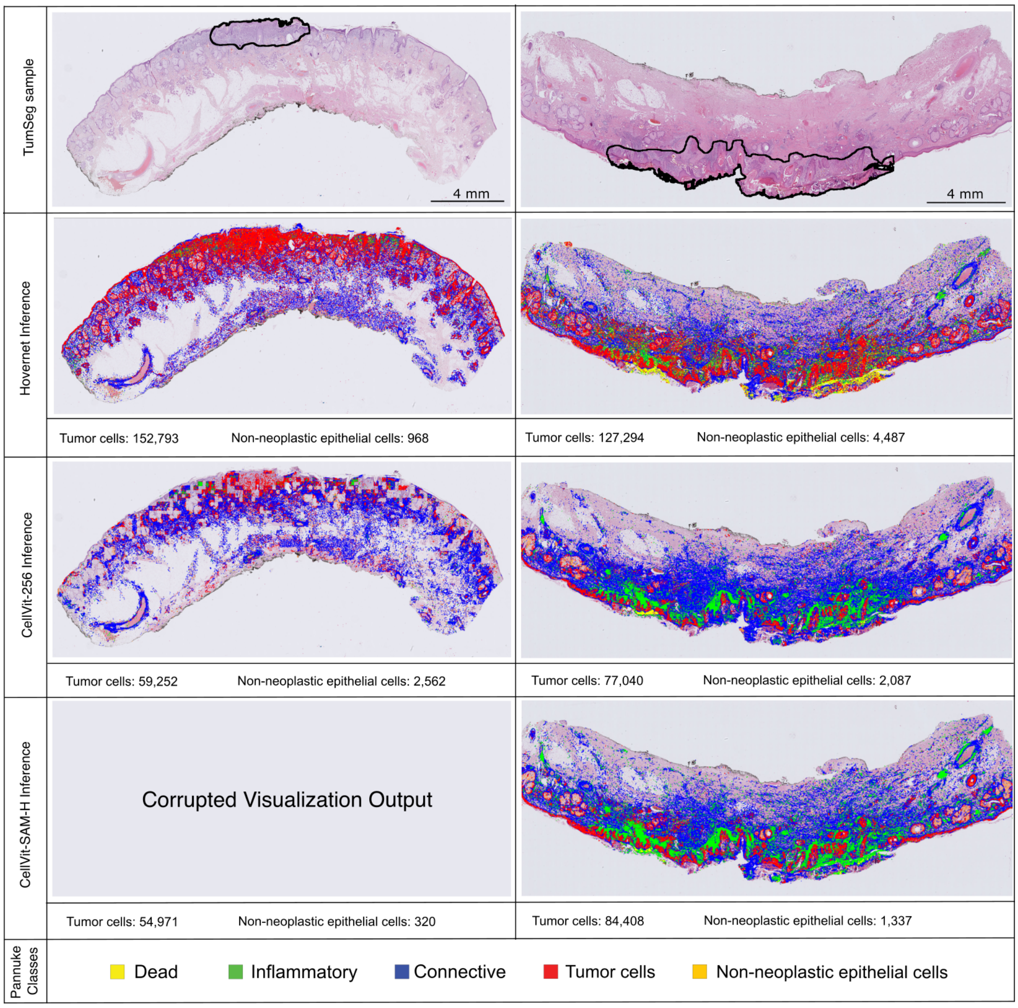

Fig A in S1 Material: Inference of pretrained Hovernet, CellViT-256 and CellVit-SAM-H on TumSeg samples. Application of state of the art models pretrained on Pannuke dataset and Hovernet, to two slides from TumSeg dataset for which tumor regions were annotated by two experts (marked in black). We display numbers of tumor and non-neoplastic epithelial cells detected by the different models. All inferences lead to prediction of tumor cells even outside tumor regions and within the healthy skin. Additionally, for some WSIs CellVit-SAM-H inference failed to generate an output visual, an issue described to sometimes occur with this model, probably when the number of cells to segment is too high.

SCC Hovernet loss functions

The overall training loss function L is a combination of loss functions as follows:

$$L = \underbrace{\lambda_{SCC_a} L_a + \lambda_{SCC_b} L_b}_{\text{HoVer Branch}} + \underbrace{\lambda_{SCC_c} L_c + \lambda_{SCC_d} L_d}_{\text{Nuclear Pixels Branch}} + \underbrace{\lambda_{SCC_e} L_e + \lambda_{SCC_f} L_f}_{\text{Nuclear Classification Branch}}$$

where L_a and L_b represent the regression loss with respect to the output of the HoVer branch, L_c and L_d represent the loss with respect to the output of the NP branch (Nuclear Pixels branch, corresponding to the Nuclear Segmentation), L_e and L_f represents the loss with respect to the output at the NC branch (Nuclear Classification branch), as already defined in [1]. Here all $\lambda_{SCC} = 1$.

$$L_a = \frac{1}{N} \sum_{i=1}^{N} \big(p_i(I; \mathbf{w_0}, \mathbf{w_1}) - \Gamma_i(I)\big)^2 \tag{1}$$

$$\begin{aligned} L_b = & \frac{1}{m} \sum_{i \in M} \big(\nabla_x \big(p_{i,x}(I; \mathbf{w_0}, \mathbf{w_1})\big) - \nabla_x \big(\Gamma_{i,x}(I)\big)\big)^2 \\ & + \frac{1}{m} \sum_{i \in M} \big(\nabla_y \big(p_{i,y}(I; \mathbf{w_0}, \mathbf{w_1})\big) - \nabla_y \big(\Gamma_{i,y}(I)\big)\big)^2 \end{aligned} \tag{2}$$

$$L_c = L_e = CE = -\frac{1}{N} \sum_{i=1}^{N} \sum_{k=1}^{K} X_{i,k}(I) \log Y_{i,k}(I) \tag{3}$$

$$L_d = L_f = DICE = 1 - \frac{2 \times \sum_{i=1}^{N} \big(Y_i(l) \times X_i(l)\big) + \epsilon}{\sum_{i=1}^{N} \big(Y_i(l)\big) + \sum_{i=1}^{N} \big(X_i(l)\big) + \epsilon} \tag{4}$$

where I input Image containing N pixels, $p_i(I; \mathbf{w_0}, \mathbf{w_1})$ regression output of HoVer branch at pixel i, w_0 and w_1 2 sets of weights. Γ_i defines the groundtruth of the horizontal and vertical distances of nuclear pixels to their corresponding centers of mass, the horizontal and vertical components of this map are denoted $\Gamma_{i,x}$ and $\Gamma_{i,y}$ respectively (see [1] for visualization and definitions). m denotes total number of nuclear pixels within the image and M denotes the set containing all nuclear pixels. ∇_x and ∇_y denote the gradient in the horizontal x and vertical y directions respectively. Finally, X denotes the branch groundtruth, Y the branch prediction, K is the number of classes and ϵ is a smoothness constant that was set to 10^{-3}.

Hyperparameters grid search for SCC Segmenter

On **Table A in S1 Material** the different hyperparameters tested are displayed. $cat - max - ratio$ corresponds to the max area ratio that could be occupied by single category for a given crop of the input image. $img - scale$ corresponds to the resizing of the image before cropping. These resizing can randomly be modified by a factor contained in [0.5, 2], following the data augmentation pipeline from the semantic segmentation library MMSegmentation [5].

Not all combinations were fully tested, some were aborted if the training loss did not decrease in the firsts epochs or if several hyperparameters in the set were already

Hyperparameters	Value Range
cat-max-ratio	[0.75, 0.85, 0.90 , 0.95, 1.0]
img-scale	[(2560, 640), (5120, 1280), (5120, 5120)]
samples-per-gpu	[4, 8]
pre-training-dataset	[ImageNet, Thomas2021 dataset [4]]

Table A in S1 Material: Hyperparameters search for SCC Segmenter

poorly performing in previous trainings. .

Set kept: {cat-max-ratio: 0.75, img-scale: (2560, 640), samples-per-gpu: 4, pre-training-dataset: ImageNet}

Probability of distance overestimation

In this paper, we make use of a search algorithm to estimate the average distance of the closest cell of a given class X - source class - to the closest cell of a given class Y - target class - inside the tumor regions. The principle is to define a search area (based on the tumor bounding box dimensions) centered around the nucleus of the source cell class and to check if any cells of the target class are inside. If it is the case, we calculate all the distances and keep the smallest one. If there is no cell inside the first search area, we increase the size of this area until we find at least one cell inside. Nevertheless, the closest distance found with this method is not always the actual closest distance, but sometimes it could be an overestimation. As shown in **Fig** B in S1 Material, sometimes a cell outside the search area can be the closest but won't be taken into account.

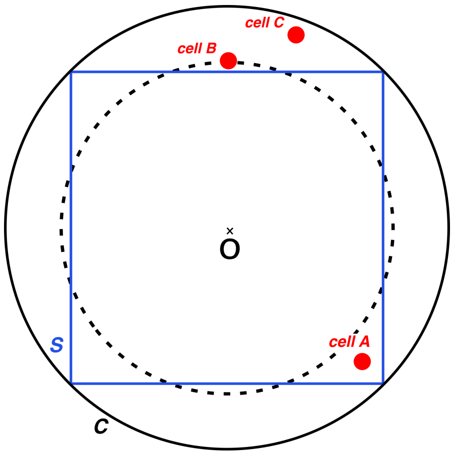

Fig B in S1 Material: Example of distance overestimation with search area being a square. Here we have 1 cell in the squared search area S and 2 cells in $(C \setminus S)$. While calculating the minimum distance of cells from the center O, if we only consider cells in S, we will take $cellA$ as being the closest cell. In reality the closest cell is $cellB$. The distance calculated will be the distance between $cellA$ and the center, and will then be higher than the real closest distance. Nevertheless, the overestimation is bounded as expressed in **Eq** 22 with $a = 1$ as we are here in the case of a squared search area.

In this supplement we show that even if such a case can occur, it is very unlikely as the number of cells increases. We simplify the problem by taking a squared search area instead of the rectangle search area that we can have in practice. The search area is based on the tumor's bounding box shape, and tumors bounding box have low aspect ratios, so taking a square search area makes it a good approximation for evaluation of error.

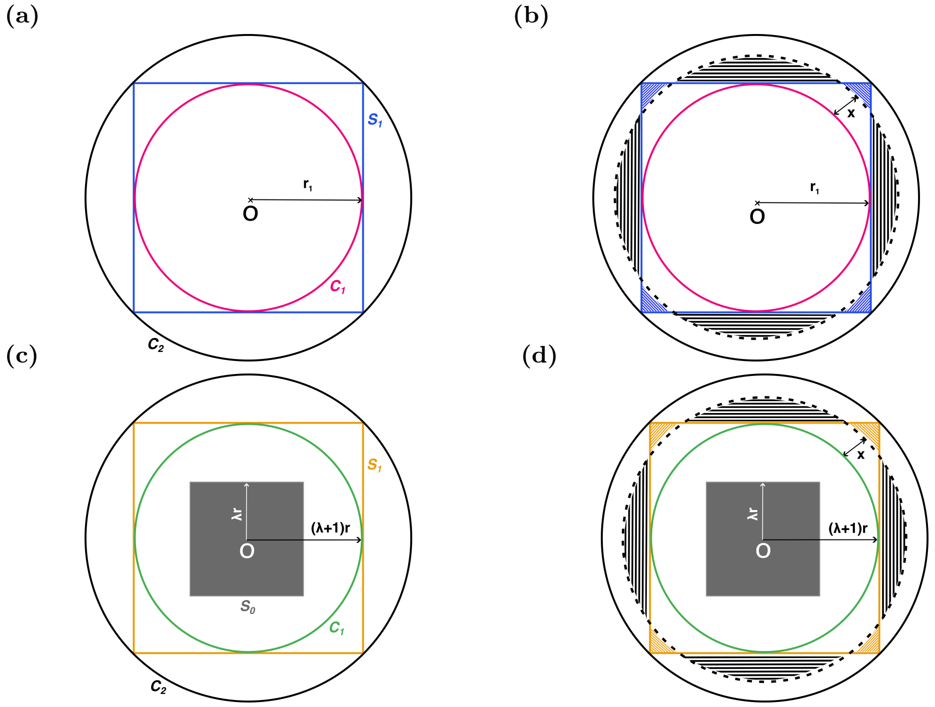

Fig C in S1 Material: Probability of having distance overestimation representations, with search area being a square. **(a)** During the first search, if the search box is a square of side lenght 2r, we could have a distance calculation error if a cell is in the blue square S_1 without being in the pink circle C_1 (then in $S_1 \setminus C_1$), and another cell is in black circle C_2 without being in in the blue square S_1 (then in $C_2 \setminus S_1$). Then the cell in $C_2 \setminus S_1$ could be missed from closest distance calculation and the cell in $S_1 \setminus C_1$ considered as the closest cell. In other cases, such as having at least one cell in S_1, no distance overestimation could be made. **(b)** To be more precise, any cell in the hatched blue area inside $S_1 \setminus C_1$ monitored by x would lead for sure to an error if another cell is in the hatched black area inside $C_2 \setminus S_1$ monitored by x . For now we focused on the probability of overestimation in the case that at least one cell is found during the first search. If no cell is found, then the calculation differs for the further searching steps as visualized in **(c)** & **(d)**. In such cases, a specific new area cannot contain any cells, as none were found during prior searches, impacting the full calculation.

We consider N cells uniformly and independently distributed inside C_2 of radius r_2 and center O. We note S_1 the inscribed square in C_2 of side length $2r_1$. We here have $r_2 = \sqrt{2}r_1$. A representation of this model is shown in **Fig** C in S1 Material **(a)**.

We are interested in computing the following conditional probability:

$$P(error_N) = P(B_N \mid A_N) \tag{5}$$

where:

$$A_N : (\text{At least one cell is in } S_1)$$

$$B_N : (\text{There is among the cells in } (C_2 \setminus S_1) \text{ a cell closer to the center } O \text{ than all cells in } S_1)$$

This probability corresponds of the probability of having an error in the distance calculation (overestimation) when at least one cell is found in the first search area C_1 of radius r_1 and center O. To compute this probability we first make use of the complementary event rule:

$$P(B_N \mid A_N) = 1 - P(\bar{B_N} \mid A_N) \tag{6}$$

where:

$$\bar{B_N} : (\text{All the cells in } (C_2 \setminus S_1) \text{ are further away to the center } O \text{ than all cells in } S_1)$$

To compute $P(\bar{B_N} \mid A_N)$, we introduce for $\forall n \in [[0, N]]$:

$$U_N(n) : (\text{Exactly } n \text{ cells are in } (C_2 \setminus S_1))$$

Using the law of total probabilities and then the definition of conditional probabilities:

$$\begin{aligned} P(\bar{B_N} \mid A_N) &= \sum_{n=0}^{N} P\big(\bar{B_N} \cap U_N(n) \mid A_N\big) \\ &= \sum_{n=0}^{N} \frac{P\big(\bar{B_N} \cap U_N(n) \cap A_N\big)}{P(A_N)} \end{aligned} \tag{7}$$

Now we note that $\forall n \in [[0, N-1]], U_N(n) \subset A_N$, so that $A_N \cap U_N(n) = U_N(n)$. We note moreover that if $n = N$, then no cells are is in the search area of S_1. So $U_N(N) \cap A_N = \emptyset$. Finally, noting that $U_N(0) \subset \bar{B_N}$ and using the definition of conditional probabilities, we have:

$$\begin{aligned} P(\bar{B_N}|A_N) &= \frac{P\big(U_N(0)\big)}{P(A_N)} + \sum_{n=1}^{N-1} \frac{P\big(\bar{B_N} \cap U_N(n)\big)}{P(A_N)} \\ &= \frac{1}{P(A_N)} \Big[P\big(U_N(0)\big) + \sum_{n=1}^{N-1} P\big(U_N(n)\big) P\big(\bar{B_N} \mid U_N(n)\big)\Big] \end{aligned} \tag{8}$$

As U_N is a binomial trial, we can calculate $P(U_N(n))$ as follows:

$$\begin{aligned} P\big(U_N(n)\big) &= \binom{N}{n} \Big(\frac{\text{area}(C_2 \setminus S_1)}{\text{area}(C_2)}\Big)^n \Big(1 - \frac{\text{area}(C_2 \setminus S_1)}{\text{area}(C_2)}\Big)^{N-n} \\ &= \binom{N}{n} \Big(1 - \frac{2}{\pi}\Big)^n \Big(\frac{2}{\pi}\Big)^{N-n} \end{aligned} \tag{9}$$

We then deduce that:

$$P\big(U_N(0)\big) = \Big(\frac{2}{\pi}\Big)^N \tag{10}$$

As A_N : (At least one cell is in S_1), $\bar{A_N}$: (No cell is in S_1) which is equivalent to $\bar{A_N}$: (All N cells are in $(C_2 \setminus S_1)$), then $\bar{A_N} = U_N(N)$ and using the complementary event rule:

$$P(A_N) = 1 - P(\bar{A_N}) \tag{11}$$
$$= 1 - P\big(U_N(N)\big)$$
$$= 1 - \Big(1 - \frac{2}{\pi}\Big)^N$$

We now set two new events introducing $d = r_1 + x$ as shown in **Fig** C in S1 Material **(b)** :

$V_N(n,d)$: (There are $N-n$ cells in S_1 and the closest cell to the origin O is at a distance d from O)

And:

$\tilde{V_N}(n,d)$: (There are $N-n$ cells in S_1 and they are all located at a distance greater than d from O)

Then, using the law of total probability for continuous univariate distributions:

$$P\big(\bar{B_N} \mid U_N(n)\big) = \int_0^{\sqrt{2}r_1} P\big(\ \bar{B_N}\ \mid \big(U_N(n) \cap V_N(n,d)\big)\big)\ \rho_n(d)\ \mathrm{d}d \tag{12}$$

where $\rho_n(d)$ is the probability density function associated to the survival function $P\big(\tilde{V_N}(n,d)|U_N(n)\big)$:

$$\rho_n(d) = -\frac{\mathrm{d}P\big(\tilde{V_N}(n,d)|U_N(n)\big)}{\mathrm{d}d}$$

If $d \leq r_1$ then $P(\bar{B_N}|(U_N(n) \cap V_N(n,d))) = 1$, leading to:

$$P\big(\bar{B}_N \mid U_N(n)\big) = \int_0^{r_1} \rho_n(d)\,\mathrm{d}d\ +\ \int_{r_1}^{\sqrt{2}r_1} P\big(\bar{B}_N \mid \big(U_N(n) \cap V_N(n,d)\big)\big)\,\rho_n(d)\,\mathrm{d}d \tag{13}$$
$$= \Big[1 - P\big(\tilde{V_N}(n,r_1) \mid U_N(n)\big)\Big]\ +\ \int_{r_1}^{\sqrt{2}r_1} P\big(\bar{B}_N \mid \big(U_N(n) \cap V_N(n,d)\big)\big)\,\rho_n(d)\,\mathrm{d}d$$

Knowing that all cells in $(S_1 \setminus C_1)$ are at a higher distance than r_1 from O, calculating $P(\tilde{V}(n,r_1)|U(n))$ gives:

$$P\big(\tilde{V_N}(n,r_1)|U_N(n)\big) = \Big(\frac{\text{area}(S_1 \setminus C_1)}{\text{area}(S_1)}\Big)^{N-n} \tag{14}$$
$$= \Big(1 - \frac{\pi}{4}\Big)^{N-n}$$

Now we consider area(*hatchblue*) and area(*hatchblack*) as defined in **Fig** C in S1 Material **(b)**. We also define the circle C_d of radius d $(d = r + x)$.

For $d \in (r_1, r_2)$:

$$\begin{aligned} P\big(\tilde{V}_N(n,d) \,\big|\, U_N(n)\big) &= \left(\frac{\text{area}(\textit{hatchblue})}{\text{area}(S_1)}\right)^{N-n} \\ &= \left(\frac{\text{area}(S_1 \setminus C_d) + \text{area}(\textit{hatchblack})}{\text{area}(S_1)}\right)^{N-n} \\ &= \left(\frac{4r_1^2 - d^2\pi + \text{area}(\textit{hatchblack})}{4r_1^2}\right)^{N-n} \end{aligned} \tag{15}$$

area($\textit{hatchblack}$) is the area of 4 circular segments of arc radius d and sagitta (d - r_1) then:

$$\begin{aligned} \text{area}(\textit{hatchblack}) &= 4A(d - r_1, d)_{CS} \\ &= 4d^2 \arccos\left(1 - \frac{d - r_1}{d}\right) - 4r_1\sqrt{d^2 - r_1^2} \\ &= 4d^2 \arccos\left(\frac{r_1}{d}\right) - 4r_1\sqrt{d^2 - r_1^2} \end{aligned} \tag{16}$$

Then:

$$\begin{aligned} P\big(\tilde{V_N}(n,d)|U_N(n)\big) &= \Big(\frac{4r_1^2 - d^2\pi + 4d^2 \arccos\left(\frac{r_1}{d}\right) - 4r_1\sqrt{d^2 - r_1^2}}{4r_1^2}\Big)^{N-n} \\ &= \Big(1 + \left(\frac{d}{r_1}\right)^2 \arccos\left(\frac{r_1}{d}\right) - \frac{d^2\pi + 4r_1\sqrt{d^2 - r_1^2}}{4r_1^2}\Big)^{N-n} \end{aligned} \tag{17}$$

So:

$$\rho_n(d) = -\frac{\mathrm{d}P\big(\tilde{V_N}(n,d)|U_N(n)\big)}{\mathrm{d}d} \tag{18}$$

$$\rho_n(d) = -(N-n)\Big(\frac{1}{r_1\sqrt{1 - \frac{r_1^2}{d^2}}} + \frac{2d \arccos\left(\frac{r_1}{d}\right)}{r_1^2} - \frac{\frac{4dr_1}{\sqrt{d^2 - r_1^2}} + 2\pi d}{4r_1^2}\Big)\Big(1 + \left(\frac{d}{r_1}\right)^2 \arccos\left(\frac{r_1}{d}\right) - \frac{d^2\pi + 4r_1\sqrt{d^2 - r_1^2}}{4r_1^2}\Big)^{N-n-1}$$

To find $P(\bar{B}|U(n), A)$ we are left with calculating $P(\bar{B}|U(n), V(n,d))$:

$$\begin{aligned} P(\ \bar{B_N}\ \ | (U_N(n) \cap V_N(n,d)) &= \Big(\frac{\text{area}(C_2 \setminus S_1) - \text{area}(\textit{hatchblack})}{\text{area}(C_2 \setminus S_1)}\Big)^n \\ &= \Big(1 - \frac{4d^2 \arccos(\frac{r_1}{d}) - 4r_1\sqrt{d^2 - r_1^2}}{(2\pi - 4)r_1^2}\Big)^n \end{aligned} \tag{19}$$

Finally:

$$\int_{r_1}^{\sqrt{2}r_1} P(\ \bar{B_N}\ \ | \big(U_N(n) \cap V_N(n,d)\big)\big)\ \rho_n(d)\ \mathrm{d}d = \tag{20}$$

$$-(N-n)\int_{r_1}^{\sqrt{2}r_1}\Big(\frac{1}{r_1\sqrt{1 - \frac{r_1^2}{d^2}}} + \frac{2d \arccos\left(\frac{r_1}{d}\right)}{r_1^2} - \frac{\frac{4dr_1}{\sqrt{d^2 - r_1^2}} + 2\pi d}{4r_1^2}\Big)\Big(1 + \left(\frac{d}{r_1}\right)^2 \arccos\left(\frac{r_1}{d}\right) - \frac{d^2\pi + 4r_1\sqrt{d^2 - r_1^2}}{4r_1^2}\Big)^{N-n-1}\Big(1 - \frac{4d^2 \arccos(\frac{r_1}{d}) - 4r_1\sqrt{d^2 - r_1^2}}{(2\pi - 4)r_1^2}\Big)^n \mathrm{d}d$$

So to sum up:

$$P(error_N) = 1 - \frac{1}{P(A_N)}\Big[P\big(U_N(0)\big) + \sum_{n=1}^{N-1} P\big(U_N(n)\big)P\big(\bar{B_N} \mid U_N(n)\big)\Big] \tag{21}$$

where $P(A_N)$ is calculated in **(11)**, $P\big(U_N(0)\big)$ is calculated in **(10)**, $P\big(U_N(n)\big)$ is calculated in **(9)** and finally $P\big(\bar{B_N} \mid U_N(n)\big)$ is developed in **(13)**, **(14)**, and **(20)**.

Now we can have a numerical resolution for the probability of error $P(error_N)$ as it was decomposed fully. N number of cells from 2 to 20 we can calculate $P(error_N)$ as shown in **Fig** D in S1 Material.

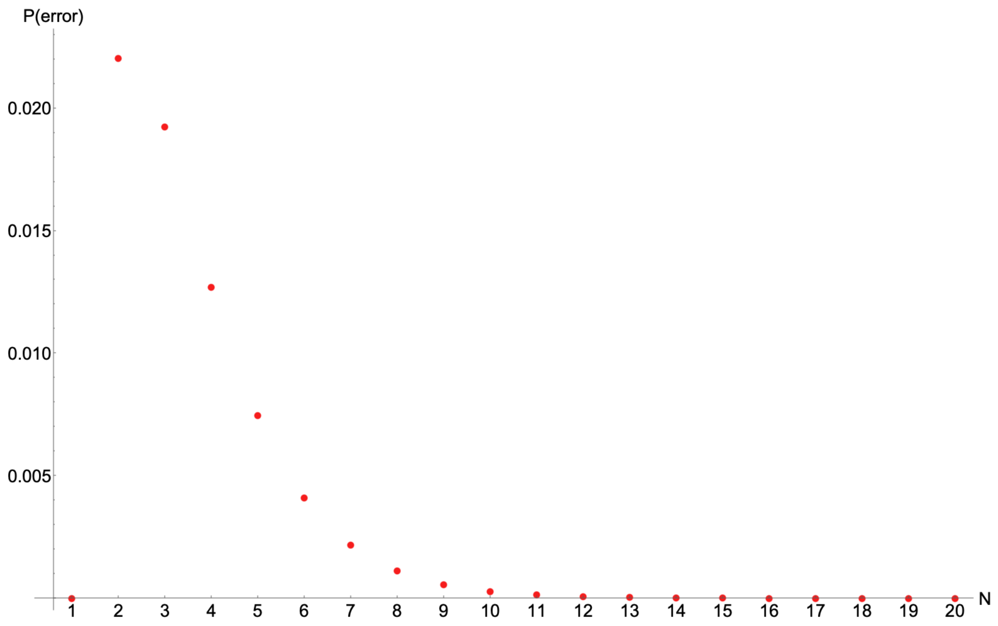

Fig D in S1 Material: Probability of overestimation of the distance calculation in the case of a squared search area. We focus in the case of the first search, if at least one cell is found in the first squared search area. We calculate $P(error_N)$ using the symbolic expression found in **Eq** 21. The probability of error is 0 for $N = 1$ as expected, because to consider the wrong cell we need at least 2 cells in C_2. For instance we have here $P(error_{N=2}) = 0.022$, $P(error_{N=10}) = 2.828\text{x}10^{-4}$ and $P(error_{N=20}) = 9.58\text{x}10^{-7}$.

To validate further our probability of distance overestimation, we can verify that the calculation does not depend of the value of r_1 (half length of search square side). Indeed, while calculating $P(error_N)$ with Wolfram Mathematica r_1 is given as a variable. This simple validation is displayed in **Fig** E in S1 Material.

We provide in our github repository (available here) a Wolfram Mathematica notebook for visualization and to calculate $P(error_N)$ for any values of N.

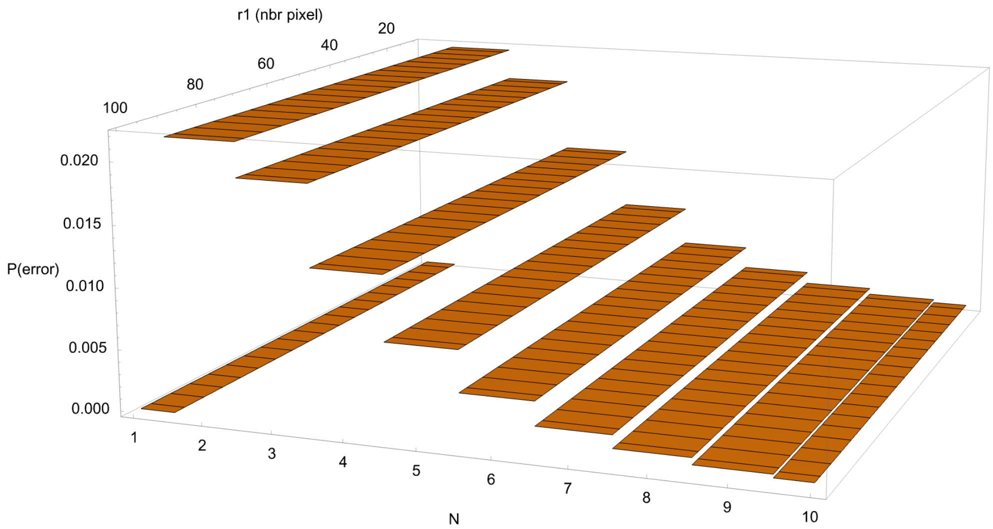

Fig E in S1 Material: Verification of independence from r_1. We verified that the probability of error is indeed independent of r_1 (half length of search square side) while calculating numerically the probability in Wolfram Mathematica. While checking the symbolic representation of $P(error_N)$ that was found, this independence is not easy to check, especially for $P(\bar{B_N} \mid U_N(n))$ (see **Eq** 13 & **Eq** 20).

Finally, we can note that the relative overestimation of the distance is bounded. Going back the the case of a rectangle search area, overestimation is bounded by the aspect ratio of the tumor bounding box:

$$d_{max-overestimation} = (\sqrt{a^2+1} - 1)\frac{w}{2} \tag{22}$$

where w is the width of the rectangle and a the aspect ratio.

We covered here the case where at least one cell is found in the first search, meaning at least one cell is in S_1. In practice, distances can be overestimated in subsequent search iterations following the first. Unfortunately the calculation will be slightly different. Visualization of the cases where no cell is found inside S_1 are available at **Fig C in S1 Material (c), Fig C in S1 Material (d)**.

List of All Features from Tissue Analyser

All the features names are organized in a json file available in the CPI image classification dataset Zenodo repository here. We calculated two types of densities. The first one corresponds to the number of cells of a given class divided by tumor regions area (in pixel) and the second one corresponds to total area of all cells of a given class that are inside tumor regions (in pixel) divided by tumor regions area (in pixel).

Ranking of Features after Cross-Validation with mRMR Selection

The ranking of features after the 3 fold cross-validation of XGBoost with mRMR feature selection is organized in a text file available in the CPI image classification dataset

Zenodo repository here.

ROC curves of classifier with best kept features for all cross-validation folds

We provide visualization of ROC curve for each splits.

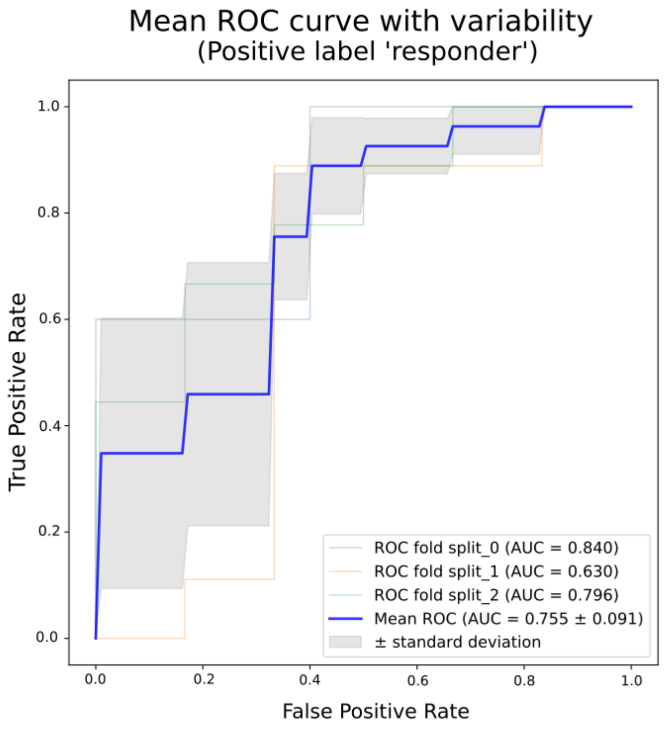

Fig F in S1 Material: Classifier cross-validation folds ROC curves.

Stain variability for the different cohorts of TumSeg dataset

We analyzed cohort-specific stain variability of TumSeg dataset by comparing RGB mean intensities between Cologne, Munich, and Bonn using the Mann–Whitney U test [6], paired with Cliff's δ [7] as a non-parametric effect size, see **Fig G in S1 Material**.

Cologne and Bonn exhibit highly similar stain characteristics across all RGB channels, with negligible effect sizes between them (e.g., $|\delta| \leq 0.09$, ns). In contrast, Munich consistently deviates from both cohorts, showing medium to large effect sizes across channels (e.g., $|\delta| = 0.31$ in the red channel, $|\delta| = 0.46$–0.55 in the green channel, and $|\delta| = 0.34$–0.57 in the blue channel). These results indicate that Munich represents a distinct staining domain. This is the reason why color normalization step implemented within SCC Segmenter training and inference is necessary [8]. As previously stated, in the pre-processing pipeline for SCC Segmenter, the images are first downsampled, then tiled into patches and the patches are normalized using mean and standard deviation of RGB pixel values of ImageNet 1K (see our github repository for implementation details).

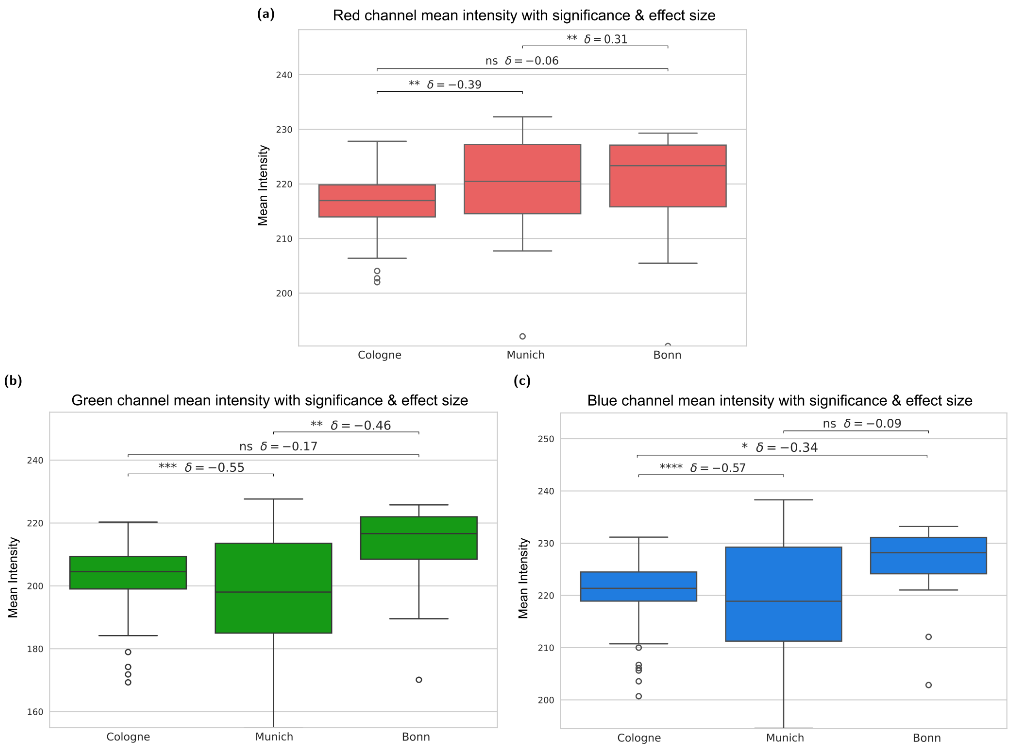

Fig G in S1 Material: Boxplots of Mean RGB pixel intensities for all slides from TumSeg dataset. We define the statistical significance of Mann-Whitney U test as the following: **ns**: $p \geq 0.05$; *****: $0.01 \leq p < 0.05$; ******: $0.001 \leq p < 0.01$; *******: $0.0001 \leq p < 0.001$; ********: $p < 0.0001$. While Cologne and Bonn slides have high similarities in their staining, Munich introduces a clear color-domain bias. The color normalization step from SCC Segmenter is necessary to uniform staining on all cohorts.

References

1. Graham, S. *et al.* Hover-net: Simultaneous segmentation and classification of nuclei in multi-tissue histology images. *Medical image analysis* **58**, 101563 (2018).
2. Hörst, F. *et al.* Cellvit: Vision transformers for precise cell segmentation and classification. *Medical image analysis* **94**, 103143 (2023).
3. Gamper, J. *et al.* Pannuke dataset extension, insights and baselines. *ArXiv* **abs/2003.10778** (2020).
4. Thomas, S. M., Lefevre, J. G., Baxter, G. W. & Hamilton, N. A. Non-melanoma skin cancer segmentation for histopathology dataset. *Data in Brief* **39** (2021).
5. Contributors, M. MMSegmentation: Openmmlab semantic segmentation toolbox and benchmark. https://github.com/open-mmlab/mmsegmentation (2020).
6. Mann, H. B. & Whitney, D. R. On a test of whether one of two random variables is stochastically larger than the other. *Annals of Mathematical Statistics* **18**, 50–60 (1947).
7. Cliff, N. Dominance statistics: Ordinal analyses to answer ordinal questions. *Psychol. Bull.* **114**, 494–509 (1993).
8. Steiner, A. P. *et al.* How to train your vit? data, augmentation, and regularization in vision transformers. *Transactions on Machine Learning Research* (2022).
