# Supplementary material for: Histo-Miner: Deep learning based tissue features extraction pipeline from H&E whole slide images of cutaneous squamous cell carcinoma
Source: PLoS Comput Biol. 2026 Jan 21;22(1):e1013907. doi: 10.1371/journal.pcbi.1013907 (PMC12854473; doi:10.1371/journal.pcbi.1013907)
Supplement: Attachment — Submitted filename: Response_to_Reviewers.pdf [file pcbi.1013907.s002.pdf]

28.11.2025,

We sincerely thank the reviewers for their thoughtful assessment of our manuscript. We greatly appreciate the constructive comments and suggestions, which have contributed significantly to strengthening the clarity and quality of the revised version.

Following reviewer comments, we re-organized some sections of the manuscript to improve readability. We gathered a new test set for SCC Segmenter evaluation as the previous evaluation on the validation set could carry biases. Our evaluation on the newly created test set, consisting of new patients, showed similar results to the previous evaluation. We added a new section to the supplement describing how color normalization is a fundamental pre-processing step for SCC Segmenter. We also explained more in detail how our dataset annotations were generated and explained the pre-processing steps for SCC Segmenter and SCC Hovernet.

Here below are our detailed responses to the Reviewers' comments:

Reviewers' comments:

Reviewer #1: Histo-Miner is a novel deep learning-based pipeline designed for the analysis of Whole-Slide Images (WSIs) of skin, specifically targeting the challenge of non-melanoma tumor cell classification, where data scarcity and high morphological similarities between tumor and healthy epithelial cells pose difficulties. The pipeline integrates convolutional neural networks and vision transformers for nucleus segmentation and classification, as well as tumor region segmentation. A key refinement step is implemented where nuclei predicted as tumor cells but located outside predicted tumor regions are reclassified as healthy epithelial, leveraging broader tissue context to overcome these morphological ambiguities. Histo-Miner introduces two new cSCC datasets and demonstrates state-of-the-art performance across multiple tasks. Importantly, it shows potential in predicting cSCC patient response to immunotherapy, highlighting key predictive cellular features such as lymphocyte percentages and granulocyte-lymphocyte ratios, and is designed for generalizability to other cancer types and datasets.

Overall, the study presents a promising and well-structured pipeline with clear potential for advancing the analysis of WSIs in cSCC and beyond. However, several details regarding the methodology and evaluation remain unclear and should be clarified to ensure the reproducibility of the study and to allow a proper assessment of the robustness and generalizability of the approach.

Comment: 2.1 Histo-Miner pipeline description. The pipeline description mentions two types of preprocessing involving downsampling, tiling, and color normalization. However, important details are missing to ensure reproducibility. Could the authors clarify whether tiles are extracted only from tissue regions or from the entire WSI? If tiles are extracted from the whole slide, are patches without tissue filtered out afterwards? The method for color normalization is not fully described, and it would be helpful to clarify how it addresses variability in staining across samples.

Response: We thank the reviewer for the suggestion and have added more explanatory details in corresponding sections of the revised manuscript. In brief, during inference, patches are taken from the full WSI, and patches with no tissue are filtered out (no pixel of tissue mask in the patch). Before the segmentation using SCC Segmenter, the mean and standard deviation of RGB pixel values of ImageNet 1K are used for normalization of images.

After verification of HoVer-Net code, there is no color normalization in the HoVer-Net pipeline (color normalization functions are in the code but finally not used). Therefore, we address staining variability using color augmentation during training: Gaussian blurs and median blurs according to the original Hovernet implementation.

We changed the Figure 1 to match this description and added the corresponding information to the manuscript.

For reproducibility, we also include our preprocessing code in our github repository.

Comment: 2.2 NucSeg and TumSeg datasets descriptions. In the dataset generation section, it is stated that class labels, segmentation masks, and tumor regions were manually annotated by two pathology experts. However, it remains unclear how the annotations from the two experts were combined, and what measures were taken to ensure consistency and reliability of the annotations across the dataset.

Response: We thank the reviewer for raising this important point. To accommodate the large scale of the dataset and annotated cells, the workload was largely distributed between both experts who used a shared set of morphological criteria and annotated separate slides. To ensure reliability, we employed supervisory quality control with a random selection of annotations spot-checked by the senior pathologist (D. Helbig). Together with additional joint review and discussion of ambiguous cases, this procedure aimed at ensuring adherence to the agreed-upon criteria and consistent definitions of tumor regions and cell classes across the entire dataset.

We moreover utilized immunohistochemistry staining on a set of test slides for multiple cell type markers to objectively validate cell lineage definitions. This provided an additional ground truth for evaluation of the experts' visual assessments and ensuring high reliability of the class labels across the entire dataset.

We have updated the Methods section (lines 195 - 197) to describe these steps in more detail.

Comment: 2.4 Training SCC Hovernet. Regarding the reported split of 5,968 patches for training and 848 patches for validation from the NucSeg dataset, could the authors clarify whether the patch division was performed at the patient level, ensuring that patches from the same patient were kept within the same split? This is important to avoid potential data leakage and overestimation of model performance.

Response: The split was not performed at the patient level; however, overlapping patches were kept within the same split. As this model serves as an intermediate component within the Histo-Miner pipeline rather than a final diagnostic classifier, our primary goal was to maximize training set diversity by including data from all patients in the training split. When we compared models performance (CellVit 256, CellViT-SAM-H and Hovernet) on NucSeg we re-trained them from scratch and used the same training and validation splits for all models.

Comment: Could the authors clarify how the validation set was used for model selection? In particular, was early stopping applied, and if so, how was it configured? Additionally, was cross-validation considered to better assess the robustness and generalizability of the results?

Response: There was no hyperparameter search performed using the validation set, and early stopping was not applied. Therefore, the validation set was not used for model selection, but solely for evaluating the trained model. The model was trained with the same hyperparameters as Hovernet original implementation (line 268).

While cross-validation could provide a more robust estimate of generalization performance, our goal here was not to re-evaluate the architecture itself — which has already been thoroughly assessed in prior work — but rather to evaluate our trained model (with its own learned weights, rather than multiple weight sets as in cross-validation) as used within the Histo-Miner pipeline.

Comment: This section reports that performance of the resulting models was highest when first pre-trained on ImageNet-21k and subsequently on the non-curated H&E nucleus dataset before fine-tuning. However, these details seem more appropriate for the sections dedicated to Results and/or Discussion rather than the methodological description

Response: This point is well taken. We included this information in the Methods section to explain the choice of our training protocol. Because the adopted 3 training sequences strategy may appear unconventional, we briefly mention that alternative strategies were tested but performed less well. We believe this clarification helps readers understand why this specific training path was selected.

<u>Comment:</u> 2.5 Training SCC Segmenter. The description states that data augmentation was applied, consisting of random resizing, random left–right flipping, and normalization of the images based on the mean and standard deviation of ImageNet-1k pixel values. However, it seems that the explanation mixes data augmentation with normalization, and this should be clarified. In addition, it would be important to specify how the data augmentation was applied (e.g., whether augmentations were always applied or with a certain probability, and whether all transformations were used simultaneously or independently.

The model was trained on 115 randomly chosen slides from the TumSeg dataset, with the remaining 29 slides used for validation. Could the authors clarify whether this split was performed at the patient level, ensuring that slides from the same patient were not included in both sets?

The accuracy estimation was performed on the validation set due to the limited number of slides and the absence of an independent test set. Could the authors clarify whether the validation set was also used to select the best model? Given the small dataset size, cross-validation is recommended to provide a more reliable estimation of model performance and to ensure that the results are not specific to a particular data split, but consistent across different splits.

<u>Response</u>: While SCC Segmenter serves solely as an intermediary component within the Histo-Miner pipeline, we agree, that it was insufficiently validated in the initial version of the manuscript and that relying solely on the validation set could lead to an overestimation of performance, especially due to the limited number of slides.

To address this concern, we now collected an additional independent test set composed of 32 slides from 25 new patients (not included in the original TumSeg training and validation splits, independent of data augmentation), from the same 3 cohorts as TumSeg. SCC Segmenter was evaluated on this new test set and achieved results comparable to those obtained on the validation set, even slightly better. The test dataset and corresponding model inferences are publicly available via our Zenodo repository (see *Data Availability*), and the manuscript's Results section has been updated accordingly (lines 500-503):

"The model achieves on the test set: segmented foreground intersection over union IoUtissue = 0.852, mean intersection over union mIoU = 0.907, accuracy of foreground segmentation Acctissue = 0.892, and average accuracy mAcc = 0.969. This is a slight improvement on the evaluation on the validation split."

The split between train and val for TumSeg were not performed at the patient level, but on the WSI level and some slides from the same patient are in both splits (8

patients have either 2 or 3 slides that are scattered in both train and val splits). It is an additional reason why a proper test set with new patients was gathered. We modified the explanation of the pre-processing to make it clearer: both SCC Segmenter and SCC Hovernet have data augmentation during training but only SCC Segmenter include color normalization (during training and before inference).

Comment: **2.6 Tissue Analyser. Regarding the cell classification refinement, it is mentioned that nuclei predicted as tumor cells outside predicted tumor regions are reclassified as healthy epithelial. This step is crucial to address the morphological similarity between tumor and healthy cells. Could the authors clarify how the robustness of this refinement is ensured against potential inaccuracies in tumor region predictions by the SCC Segmenter? For instance, if a region is incorrectly classified as non-tumor, how does this affect the reclassification of genuinely tumor nuclei within that region? Are confidence thresholds or probability scores used to mitigate such errors?**

Response: We thank the reviewer for this relevant comment. One of the major limitations of applying existing cell type classification tools to non-melanoma skin cancer is the near universal misclassification of non-tumor epithelial as cancer cells (see Suppl. Fig. 1). We therefore designed a two-step approach to mitigate this problem. However, as correctly pointed out by the reviewer, it comes at the cost of potential false negative predictions of tumor cells. In a region incorrectly classified as non-tumor, tumor cells within that area would be incorrectly labeled as healthy epithelial cells. On the cell-type level there is no post-processing step to correct such misclassifications. Instead, we focused on optimizing tumor area detection.

Our initial evaluations of tumor segmentation shows that tumor region misclassification errors are limited. We have now moreover performed additional validation in a newly annotated set of WSI that were not part of the previous version. Here, we observe foreground intersection over union of 0.852, mean intersection over union of 0.907, accuracy of foreground segmentation of 0.892, and average accuracy of 0.969. Overall, compared to the state-of-the-art method (see Supplementary Fig. 1), our approach considerably reduces the widespread misclassification of healthy epithelial cells as tumor cells and represents a substantial improvement over prior approaches.

We have added these explanations to discussion in the revised manuscript (lines 628-631).

Comment: 2.7 Feature selection The subsection on feature selection in the case study appears under Methods but mixes methodological description with reporting of results. It would be helpful if the authors clearly separate the description of the feature selection procedure from the results obtained, to improve clarity and reproducibility.

Response: We thank the reviewer for pointing this out. We moved the paragraph about the low-performing feature selection methods from the method section to the result section.

Reviewer #2: This manuscript presents Histo-Miner, a deep learning-enabled pipeline for analyzing whole-slide images (WSIs) of cutaneous Squamous Cell Carcinoma (cSCC). The pipeline incorporates a trained convolutional neural network for the segmentation and classification of nuclei and a trained vision transformer for the segmentation of tumor areas. After validating the accuracy of Histo-Miner with manually annotated class labels and segmentation masks, the framework creates a compact feature vector summarizing tissue morphology and cellular interactions. To demonstrate its clinical applicability, the authors utilized this pipeline to predict immunotherapy response in 45 cSCC patients based on their pre-treatment WSIs.

Overall, the manuscript is well-written. However, I would like to suggest the following major revisions before getting it published:

Comment: On Page 2, line 70, it would be beneficial if the authors could provide a few references for this claim.

Response: We thank the reviewer for pointing this out. In fact, it seems that this issue has not been well-described in the literature yet despite the considerable misclassification of state-of-the-art models in our own work (see Suppl. Fig. S1). Lacking a substantial reference, we have therefore removed this claim from the introduction and moved this finding to the results/discussion parts of our manuscript.

Comment: Zenodo repository links in supplementary materials are not functional. I believe they should be formatted as https://zenodo.org/records/13986860

Response: We updated the URLs.

<u>Comment:</u> On Page 6, lines 163, 179, and 180, the term "resolution" was used instead of "magnification" when referring to microscope magnification settings. It would be helpful if the authors either clarify the actual resolutions (not magnifications) or consistently use the term "magnification" throughout these lines.

<u>Response:</u> We changed “resolution” to “magnification” as the term is indeed the correct one to use here.

<u>Comment:</u> I could not find evidence that the developed method was compared against blind test samples (WSIs). If this comparison was not performed, would it be possible to analyze the accuracy of the technique using blind test samples?

<u>Response:</u> We thank the reviewer for raising this point. In our study, the evaluation was indeed performed on blind WSIs.

We used a 3 fold cross-validation strategy in which the data was split into 2 sets, train and test. The train set contains 2/3rd of the data and the test 1/3rd. The test and train split change across runs. The test set contained only feature vectors from whole-slide images (and corresponding patients) that were not included in the training set. The test split served us to assess the final classifier and included WSIs that were not used at any stage of training or hyperparameter tuning, ensuring an unbiased, blind evaluation of the method’s performance.

We have updated the method section to make this clearer. We also moved the paragraph about the low-performing feature selection methods from the method section to the result section to make the method section easier to read.

<u>Comment:</u> On Page 4, line 123, the authors mention training and testing the models using manually annotated datasets. It would be valuable if the authors could elaborate on the potential problems of manual labeling and their impact on model accuracy in the discussion section.

<u>Response:</u> We added the following sentences to our limitations paragraph in the discussion section: “*A limitation of our approach is the requirement of expert-annotated samples (tumor regions, nucleus boundaries, and cell classes) that serve as ground truth for training and testing. It may contain human errors and introduce subjective biases that the models ultimately learn to replicate.* ”.

Reviewer #3: Summary
The paper introduces a deep learning-based pipeline for the analysis of skin Whole-Slide Images (WSIs), called Histo-Miner. To this aim, the Authors generated new datasets of cutaneous Squamous Cell Carcinoma (cSCC). Histo-Miner is presented as a good predictor for nucleus segmentation, nucleus classification, and tumor region segmentation, achieving results comparable to the state of the art, with improvements in the classification of healthy tissue. Moreover, the work also introduces the use of the tool with a predictive objective for immunotherapy response.

Overall Quality Statement
The work appears to be of very high quality. It presents an interesting tool with strong potential both in image recognition and in its integration with mathematical models, particularly regarding response prediction. The material is clearly presented, with a good balance between text and supplementary material. The flow of the exposition is clear and well documented, including information on the pipeline, practical examples, validation of results, and presentation of possible further applications. Although my expertise is more on the side of application than on the technical aspects of the employed methodologies, I consider this manuscript to be of high value, both for its technical quality and for the usefulness of its content.

Minor Observations

R133:
"After determining tumor areas using SCC Segmenter, the results of the SCC Hovernet cell nuclei classification are updated to add a new cell class as follows: all the nuclei predicted as tumor cells outside of the predicted tumor regions are reclassified as healthy epithelial."
<u>Comment:</u> Could you please discuss the potential risk of omitting information related to invasive tissues or metastatic initiation? Have you checked whether there is any correspondence, when analyzing unresponsive tissues, with tumors showing a large number of cells outside predicted tumor regions?

<u>Response:</u> This point is well taken. A major challenge for cell type classification in non-melanoma skin cancer like cSCC is the high morphological variability of epithelial cells of healthy, i.e. non-tumor, skin and their morphological similarity to tumor cells. This leads to the very high mis-classification of non-tumor epithelial cells as tumor cells observed with three pre-trained state-of-the-art models that essentially reach no distinction of tumor from non-tumor epithelial cells (see Suppl. Fig. 1). Our two-stage approach provides considerably better performance by identifying tumor

areas including larger regions and nests of tumor cells. On the other hand, due to our design, it cannot be excluded that individual cancer cells outside of tumor regions are misclassified. We have described this issue in the discussion as a potential limitation of our approach.

Our use-case highlights that our approach allows us to make accurate predictions in a clinically relevant scenario and generate explainability that seems biologically plausible considering the existing literature on immunotherapy response. It thereby presents a relevant advance for non-melanoma skin cancers compared to existing cell type classification models tested within our study.

R144:
"For every pair of cell classes X and Y, we also calculate the average distance of the closest cell of class Y to a cell of class X inside the tumor regions."
<u>Comment:</u> Since later in the manuscript the Authors state that they evaluate local cell densities, I would find it more natural to suggest considering the average density instead (i.e., the average number of cells of class Y around cells of class X within a given sensing radius). This approach would also help address boundary effects, as density can be evaluated relative to the proportion of the sphere lying within the tissue, thereby avoiding singularities. Could you please provide an explanation for this choice?

<u>Response:</u> We did not evaluate local cell densities, but calculated average closest distance and global cell densities. This point was not clear enough so we added explanations about density calculations in the supporting information. We evaluated cell densities at a more global scale - inside tumor regions. The cell densities (list of features is provided in https://zenodo.org/records/15836085) are calculated as the number of cells of a given class divided by tumor regions area (in pixel) as well as the total area of all cells of a given class inside tumor regions (in pixel) divided by tumor region area (in pixel). In these cases there is no such boundary effects, for instance if we take number of cells of a given class divided by tumor regions area, we will only consider cell inside tumor regions. The distance calculation is local, it quantifies how close cells of class X are from cells of class Y.

Additionally, using a radius to determine number of cells within this radius could mean new calculations of distances. As a reminder, distance calculations, even optimized, needs to be limited to a certain numbers to be able to calculate them in a reasonable amount of time (as seen in Methods). For instance if one wants to calculate distances of all cells to each other and then check if the distance between cell A and cell B is below the given radius, the number of distances to calculate would be C(500.001, 2) for a slide of 500.000 cells (so >10^11 distances to calculate). It is why other strategies, such as the search squared area described in the paper, are needed.

R176:
“To build a tumor segmenter algorithm, we additionally assembled 144 WSIs of 125 cSCC patients from 3 medical centers – Bonn, Cologne, and Munich.”
<u>Comment:</u> It would be useful to include information about data uniformity, since the dataset originates from different centers.

Response: This is a relevant issue. To further assess data uniformity, we analyzed cohort-specific stain variability of TumSeg dataset by comparing RGB mean intensities between Cologne, Munich, and Bonn using the Mann–Whitney U test, paired with Cliff’s delta as a non-parametric effect size, see Supplementary Fig. 7.

Cologne and Bonn exhibit highly similar stain characteristics across all RGB channels, with negligible effect sizes between them. In contrast, Munich consistently deviates from both cohorts, showing medium to large effect sizes across channels.This is the reason why color normalization step implemented within SCC Segmenter training and inference is necessary to bring all cohorts to the same color range (Steiner et al., 2022)**.**

We created a new section in supplementary material: *Stain variability for the different cohorts of TumSeg dataset* in which we added this information and the corresponding boxplots.

R220:
“We list loss functions in Supplementary Eq. 1–4.”
<u>Comment:</u> While it is functional to place technical information in the supplementary material, a short descriptive summary in the main text would be valuable. For instance, a brief explanation of which elements the loss functions account for, and whether these are considered as absolute values or in terms of slope.

Response: This point is well taken and we have now added a brief explanation of the loss function to the main text that outlines what elements it accounts for. The global loss function is an addition of 3 losses for each of the 3 branches of the Hovernet Model. The 3 branches account for the nuclear pixel classification task, the binary segmentation of nucleus, and the horizontal and vertical distance maps used to separate touching instances. To enhance clarity and readability, we added a more detailed explanation of the loss function’s elements to the supplementary material. The description specifies if a given term is a gradient or a scalar.

R253:
“The accuracy estimation was performed on the validation set due to the limited number of slides and lack of an independent test set.”
<u>Comment:</u> Since accuracy was evaluated on the validation set rather than an independent test set, the reported performance may be overestimated. The limited number of slides further reduces the reliability of this estimation. I would suggest either cross-validation or the use of an external validation cohort to better assess generalizability. Could you clarify whether the lack of independence arises from the nature of the dataset itself, or from reliance on data augmentation?

Response: We thank the reviewer for this constructive comment. While SCC Segmenter serves solely as an intermediary component within the Histo-Miner pipeline, we agree, that it was insufficiently validated in the initial version of the manuscript and that relying solely on the validation set could lead to an overestimation of performance, especially due to the limited number of slides.

To address this concern, we now collected an additional independent test set composed of 32 slides from 25 new patients (not included in the original TumSeg training and validation splits, independent of data augmentation), from the same 3 cohorts as TumSeg. SCC Segmenter was evaluated on this new test set and achieved results comparable to those obtained on the validation set, even slightly better. The test dataset and corresponding model inferences are publicly available via our Zenodo repository (see *Data Availability*), and the manuscript’s Results section has been updated accordingly (lines 500-503):

“The model achieves on the test set: segmented foreground intersection over union IoUtissue = 0.852, mean intersection over union mIoU = 0.907, accuracy of foreground segmentation Acctissue = 0.892, and average accuracy mAcc = 0.969. This is a slight improvement on the evaluation on the validation split.”

As a comparison:

	Old evaluation on Val split	New evaluation on new Test split
IoU_{tissue}	0.800	0.852
mIoU	0.884	0.907
Acc_{tissue}	0.837	0.892
mAcc	0.957	0.969
